# Gold-catalyzed reaction of oxabicyclic alkenes with electron-deficient terminal alkynes to produce acrylate derivatives

**DOI:** 10.3762/bjoc.9.233

**Published:** 2013-10-01

**Authors:** Yin-wei Sun, Qin Xu, Min Shi

**Affiliations:** 1 Key Laboratory for Advanced Materials and Institute of Fine Chemicals, School of Chemistry & Molecular Engineering, East China University of Science and Technology, 130 MeiLong Road, Shanghai 200237, People’s Republic of China,; 2 State Key Laboratory of Organometallic Chemistry, Shanghai Institute of Organic Chemistry, Chinese Academy of Sciences, 354 Fenglin Road, Shanghai 200032, People’s Republic of China

**Keywords:** gold catalysis, oxabicyclic alkenes, substituted acrylates

## Abstract

Oxabicyclic alkenes can react with electron-deficient terminal alkynes in the presence of a gold catalyst under mild conditions, affording the corresponding addition products in moderate yields. When using alkynyl esters as substrates, the (*Z*)-acrylate derivatives are obtained. Using but-3-yn-2-one (ethynyl ketone) as a substrate, the corresponding addition product is obtained with (*E*)-configuration. The proposed mechanism of these reactions is also discussed.

## Introduction

**Scheme 1:**
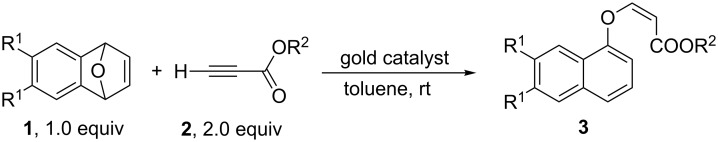
Gold-catalyzed reactions of oxabicyclic alkenes with electron-deficient terminal alkynes.

Oxabicyclic alkenes are common intermediates in organic synthesis since these compounds can be easily prepared and have a high reactivity for further transformations [1–8]. For example, they are often used to construct substituted tetrahydronaphthalene skeletons in the presence of metal catalysts such as Pd [9–10], Ir [11–15], Rh [16–21] and Cu [22]. However, their reactivity in the presence of gold catalysts has been rarely reported [23]. It is well known that gold catalysts have different catalytic abilities compared with other transition metals [24]. Moreover, gold-catalyzed chemical transformations have made significant progress during the last 5 years [25–56]. Many gold complexes have been proved to be efficient catalysts in C–C [33–48] bond or C–X (X = heteroatom) [49–56] bond forming reactions. Our group has a long-standing interest in gold-catalyzed C–C [57–61] or C–X bond [62–67] formation reactions. So far, we have reported a variety of gold-catalyzed intramolecular rearrangements with highly strained small rings for C–C or C–X bond formations [57–59,62–68]. Based on these previous findings, we envisaged that oxabicyclic alkenes could also react with electron-deficient alkynes in the presence of gold catalysts to generate a new C–C or C–O bond thereby releasing the oxabicyclic alkenes of their ring strain. In this paper, we report the formation of (*Z*)-acrylate derivatives in the gold catalyzed intermolecular reaction of oxabicyclic alkenes with electron-deficient terminal alkynes under mild conditions [69–76] (Scheme 1).

## Results and Discussion

**Figure 1:**
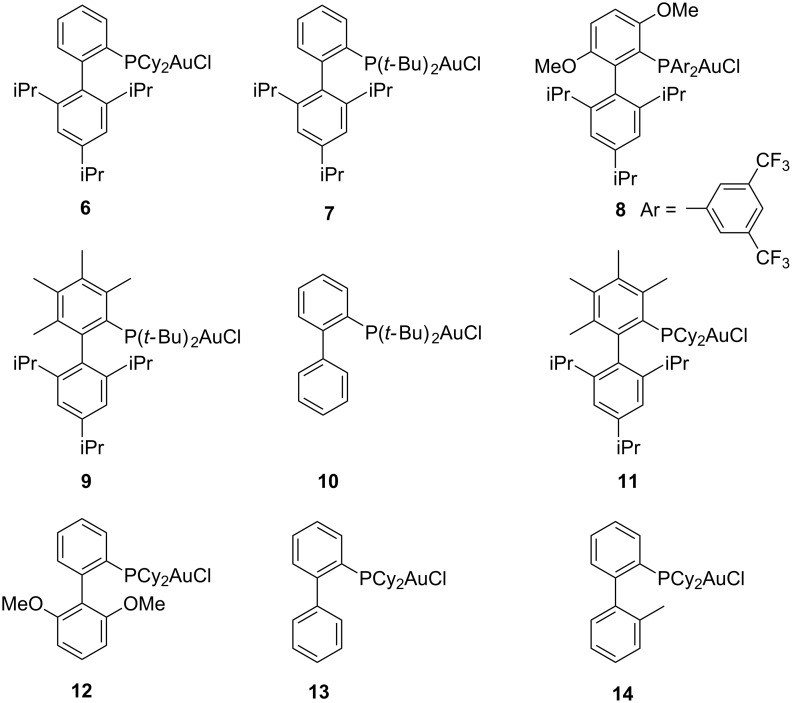
Gold complexes used in this reaction.

To generate a new C–O bond in the reaction of oxabicyclic alkene **1a** with electron-deficient terminal alkyne **2a**, we first used PPh_3_AuCl as a catalyst, AgSbF_6_ as an additive, and toluene as a solvent to examine the reaction outcome. Acrylate derivative **3a** was formed with (*Z*)-configuration in 11% yield (Table 1, entry 1). In this reaction, naphthalen-1-ol was also obtained with 44% yield as the major product. The usage of IPrAuCl, dppb(AuCl)_2_, (*p*-FC_6_H_4_)_3_PAuCl, DPE-phos(AuCl)_2_, Me_3_PAuCl and Cy_3_PAuCl as gold catalysts did not significantly improve the yield of **3a** (Table 1, entries 2–7). In these cases, the maximum yield of **3a** was 34% when the gold complex Cy_3_PAuCl coordinated by an electron-rich phosphine ligand was used as a catalyst (Table 1, entry 7). In order to further improve the yield of **3a**, we employed gold complex **6** (Figure 1) coordinated by a sterically bulky and electron-rich biaryl phosphine-type ligand as a catalyst, affording **3a** in 40% yield (Table 1, entry 8). In the absence of AgSbF_6_, no reaction occurred (Table 1, entry 9). The usage of AgSbF_6_ as a catalyst produced naphthalen-1-ol (**5**) as the major product (Table 1, entry 10). Next, we further screened the reaction conditions with gold complex **6** as a catalyst. When using AgOTs or CF_3_CO_2_Ag as a silver additive, we did not obtain any of the desired products (Table 1, entries 12 and 13), whereas the usage of AgNTf_2_ as a silver additive afforded **3a** in 32% yield (Table 1, entry 11). AgOTf was not an effective silver additive, giving **3a** in 20% yield (Table 1, entry 14). Utilization of the already prepared electrophilic cationic phosphinogold(I) complexes XPhosAuNTf_2_ and XPhosAu(MeCN)SbF_6_ as gold catalysts slightly increased the yield of **3a** to 33% and 45% yields, respectively (Table 1, entries 15 and 16). The examination of solvent effects revealed that toluene was the best solvent (Table 1, entries 17–22). Adding 4 Å MS into the reaction system, **3a** was obtained in only 10% yield (Table 1, entry 23).

**Table 1:** Initial screening of the reaction conditions.

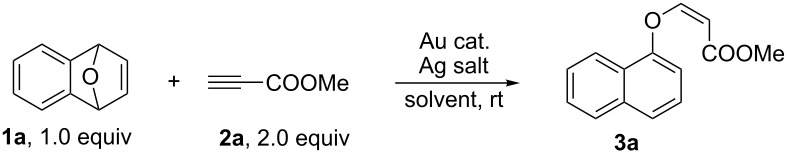				

Entry^a^	Au cat.	Ag salt	Solvent	Yield^b^ (%) **3a**

1^c^	Ph_3_PAuCl	AgSbF_6_	toluene	11
2	IPrAuCl	AgSbF_6_	toluene	19
3	dppb(AuCl)_2_	AgSbF_6_	toluene	trace
4	(*p*-FC_6_H_4_)_3_PAuCl	AgSbF_6_	toluene	26
5	DPE-phos(AuCl)_2_	AgSbF_6_	toluene	N.R.
6	Me_3_PAuCl	AgSbF_6_	toluene	N.R.
7	Cy_3_PAuCl	AgSbF_6_	toluene	34
8	**6**	AgSbF_6_	toluene	40
9	**6**	–	toluene	N.R.
10^c^	None	AgSbF_6_	toluene	trace
11	**6**	AgNTf_2_	toluene	32
12	**6**	AgOTs	toluene	N.R.
13	**6**	CF_3_COOAg	toluene	N.R.
14	**6**	AgOTf	toluene	20
15	XPhosAuNTf_2_	–	toluene	33
16	XPhosAu(MeCN)SbF_6_	–	toluene	45
17	XPhosAu(MeCN)SbF_6_	–	CH_3_CN	trace
18	XPhosAu(MeCN)SbF_6_	–	CH_3_NO_2_	N.R.
19	XPhosAu(MeCN)SbF_6_	–	Et_2_O	N.R.
20	XPhosAu(MeCN)SbF_6_	–	THF	complex
21	XPhosAu(MeCN)SbF_6_	–	DCM	25
22	XPhosAu(MeCN)SbF_6_	–	DCE	28
23^d^	XPhosAu(MeCN)SbF_6_	–	toluene	10

^a^The reaction was carried out on a 0.2 mmol scale in solvent (1.0 mL). The ratio of **1a**/**2a** was 1:2. ^b^Yield determined by ^1^H NMR by using 1-iodo-2-methoxybenzene (**4**) as an internal standard. ^c^Naphthalen-1-ol (**5**) was the major product. ^d^50 mg of 4 Å MS was added to the reaction system.

Since the yield of **3a** was still low, we next tried to improve the yield of **3a** by deploying different ligands, Ag salts, solvents and temperature. The results are summarized in Table 2. At first, we examined many other gold(I) phosphane complexes with dialkylbiarylphosphane ligands (Figure 1) by using AgSbF_6_ as an additive and toluene as a solvent. No reaction occurred when gold(I) phosphane complexes **8–10** (Figure 1) were used as catalysts under identical conditions (Table 2, entries 2–4). Furthermore, the usage of gold(I) phosphane complexes **7**, **11**, **13** and **14** (Figure 1) as catalysts gave **3a** in 10–29% yields (Table 2, entries 1, 5, 7 and 8). Gold complex **12** (Figure 1) with an electron-rich biphenylphosphine ligand was identified as the best catalyst, giving **3a** in 67% yield (Table 2, entry 6). We attempted to further optimize the reaction conditions by using SPhosAuCl **12** as a catalyst and AgNTf_2_ or AgSbF_6_ as an additive and obtained **3a** in 66% and 67% yields, respectively (Table 2, entries 6 and 9). However, the use of AgOTf or AgBF_4_ as an additive afforded **3a** in 37% and 11% yields, respectively (Table 2, entries 10 and 11). Employment of the prepared electrophilic cationic phosphinogold(I) complex SPhosAu(MeCN)SbF_6_ as a catalyst gave **3a** in 78% NMR based yield and 67% isolated yield (Table 2, entry 12). The phosphinogold(I) complex SPhosAuNTf_2_ produced **3a** in 53% yield under the standard conditions (Table 2, entry 13). The examination of solvent effects disclosed that toluene was the best solvent (Table 2, entries 14–16). Either increasing or decreasing the reaction temperature did not further improve the reaction outcome (Table 2, entries 17–20). Careful screening of the reaction conditions led to the conclusion that the reaction should be carried out in toluene at room temperature with SPhosAu(MeCN)SbF_6_ as the catalyst (Table 2, entry 12).

**Table 2:** Further screening of the reaction conditions.

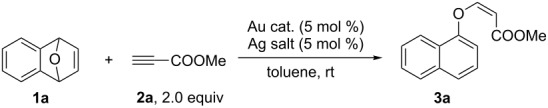					

entry^a^	Au cat.	Ag salt	solvent	*T* (°C)	Yield^b^ (%) **3a**

1^c^	**7**	AgSbF_6_	toluene	rt	11
2	**8**	AgSbF_6_	toluene	rt	N.R.
3	**9**	AgSbF_6_	toluene	rt	trace
4	**10**	AgSbF_6_	toluene	rt	N.R.
5	**11**	AgSbF_6_	toluene	rt	10
6	**12**	AgSbF_6_	toluene	rt	67
7	**13**	AgSbF_6_	toluene	rt	20
8	**14**	AgSbF_6_	toluene	rt	29
9	**12**	AgNTf_2_	toluene	rt	66 (59)^c^
10	**12**	AgOTf	toluene	rt	37
11	**12**	AgBF_4_	toluene	rt	11
12	SPhosAu(MeCN)SbF_6_	–	toluene	rt	78 (67)^c^
13	SPhosAuNTf_2_	–	toluene	rt	53
14	SPhosAu(MeCN)SbF_6_	–	DCM	rt	49
15	SPhosAu(MeCN)SbF_6_	–	DCE	rt	55
16	SPhosAu(MeCN)SbF_6_	–	CHCl_3_	rt	45
17^d^	SPhosAu(MeCN)SbF_6_	–	toluene	0	50
18	SPhosAu(MeCN)SbF_6_	–	toluene	40	45
19	SPhosAu(MeCN)SbF_6_	–	toluene	10	59
20	SPhosAu(MeCN)SbF_6_	–	toluene	30	70

^a^The reaction was carried out on a 0.2 mmol scale in solvent (1.0 mL) and the ratio of **1a**/**2a** was 1/2. ^b^Yield determined by ^1^H NMR by using 1-iodo-2-methoxybenzene **4** as an internal standard. ^c^Isolated yield in parentheses.

**Scheme 2:**
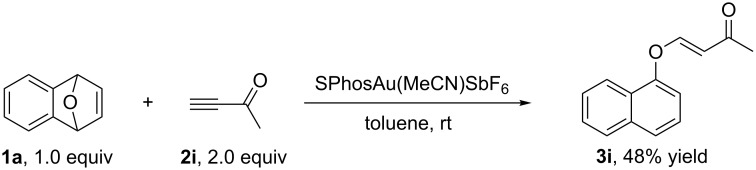
The reaction with terminal alkyne **2i** as a substrate.

Having identified the optimal conditions, we next examined the substrate scope of this reaction. We found that only the usage of ethynylbenzene and dimethyl but-2-ynedioate as substrates did not afford any of the desired products (Table 3, entries 6 and 9). In all other cases, the reactions proceeded smoothly to give the desired products in moderate to good yields (Table 2, entries 1–5, 7 and 8). The introduction of electron-donating substituents on the benzene ring impaired the reaction outcome (Table 3, entries 1 and 7). Increasing the steric hindrance of the ester group improved the yields of **3** (Table 3, entries 4 and 5). The usage of but-3-yne-2-one (terminal alkyne ketone) **2i** as a substrate gave the corresponding **3i** with (*E*)-configuration in 48% yield (Scheme 2).

**Table 3:** Substrate scope of the reactions with SPhosAu(MeCN)SbF_6_ as a catalyst.

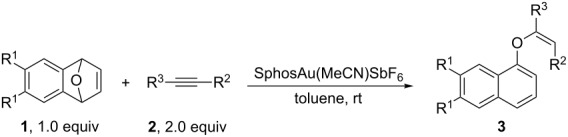			

entry^a^	R^1^	R^2^, R^3^	Yield^b^ (%) **3**

1	Me	COOMe, H	**3b**, 42
2	F	COOMe, H	**3c**, 66
3	Br	COOMe, H	**3d**, 76
4	H	COOEt, H	**3e**, 54
5	H	COO*t-*Bu, H	**3f**, 84
6	H	Ph, H	trace
7	Me	COO*t*-Bu, H	**3g**, 58
8	Br	COO*t*-Bu, H	**3h**, 72
9	H	COOMe, COOMe	N.R.

^a^The reaction was carried out on a 0.2 mmol scale in solvent (1.0 mL). The ratio of **1**/**2** was 1/2. ^b^Isolated yield.

**Scheme 3:**
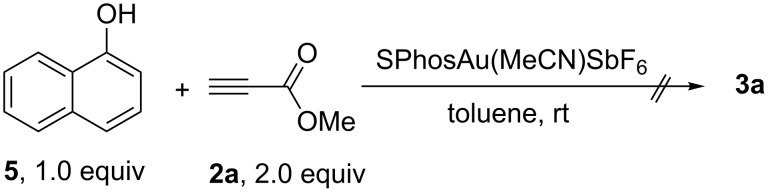
The reaction with naphthalen-1-ol (**5**) as a substrate.

Since naphthalene-1-ol (**5**) was obtained in this reaction, we used naphthalene-1-ol (**5**) as a substrate and carried out the reaction under the optimal conditions to clarify whether the reaction proceeded through naphthalene-1-ol. The formation of **3a** could not be observed, suggesting that naphthalene-1-ol is not the intermediate in this reaction (Scheme 3).

**Scheme 4:**
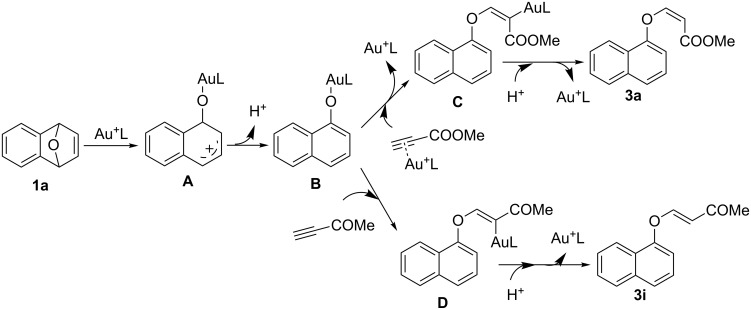
The proposed mechanism for Au(I)-catalyzed reaction.

Based on the previously established mechanistic model [23,77], we propose the following pathway for the formation of acrylate derivatives **3a** and **3i** (Scheme 4). In the presence of cationic phosphinogold(I) complex, a cationic intermediate **A** is formed by a regioselective opening of the oxygen bridge in substrate **1a**. Intermediate **A** releases a proton to afford intermediate **B**. Intermediate **B** attacks methyl propiolate, which is activated by the gold catalyst, to generate gold vinyl complex **C**. In intermediate **C**, the ester group and the naphthalene ring are on the same side, yielding the final product **3a** with (*Z*)-configuration via protodeauration. The alkyne ketone **3i** is more electron-deficient and more reactive than methyl propiolate and it is more difficult to coordinate by the gold complex. Therefore, with alkyne ketone **2i** as a substrate, intermediate **B** attacks non-coordinated alkyne ketone **2i** in a *cis*-addition manner to generate gold vinyl complex **D**. In intermediate **D**, the carbonyl group and the naphthalene ring are on opposite sides, affording product **3i** with (*E*)-configuration by protodeauration.

## Conclusion

In summary, we have developed a novel method to synthesize acrylate derivatives from oxabicyclic alkenes and electron-deficient terminal alkynes in toluene in moderate to good yields in the presence of the gold catalyst SPhosAu(MeCN)SbF_6_ under mild conditions. Efforts are in progress to elucidate the mechanistic details of this reaction and to disclose its scope and limitations.

## Experimental

### General remarks

Dichloromethane was freshly distilled from calcium hydride; THF and toluene were distilled from sodium under an argon atmosphere. ^1^H NMR, ^13^C NMR and ^19^F NMR spectra were recorded on a Bruker AM-400 spectrometer. Infrared spectra were recorded on a Perkin-Elmer PE-983 spectrometer with absorption in cm^−1^. Flash column chromatography was performed by using 300–400 mesh silica gel. For thin-layer chromatography (TLC), silica gel plates (Huanghai GF254) were used. Mass spectra were recorded by ESI, and HRMS were measured on a HP-5989 instrument.

### General procedure for the reaction catalyzed by Au(I) catalysts

Into an oven-dried reaction flask under Ar gas protection was added oxabicyclic alkene (0.2 mmol), Au catalyst (0.001 mmol), methyl propiolate (0.4 mmol) and toluene (1.0 mL). The reaction mixture was stirred at room temperature normally overnight. After complete consumption of the starting materials, monitored by TLC, the solvent was removed under reduced pressure and the residue was purified by flash column chromatography.

## Supporting Information

File 1 Experimental procedures and characterization data of compounds.
