# Supplementary material for: Gold-catalyzed reaction of oxabicyclic alkenes with electron-deficient terminal alkynes to produce acrylate derivatives
Source: Beilstein J Org Chem. 2013 Oct 1;9:1969–76. doi: 10.3762/bjoc.9.233 (PMC3817539; doi:10.3762/bjoc.9.233)
Supplement: File 1 — Experimental procedures and characterization data of compounds. [file Beilstein_J_Org_Chem-09-1969-s001.pdf]

Supporting Information

for

Gold-catalyzed reaction of oxabicyclic alkenes with electron-deficient terminal alkynes to produce acrylate derivatives

Yin-wei Sun[1], Qin Xu*[1] and Min Shi*[1,2§]

Address: [1]Key Laboratory for Advanced Materials and Institute of Fine Chemicals, School of Chemistry & Molecular Engineering, East China University of Science and Technology, 130 MeiLong Road, Shanghai 200237, People's Republic of China, and [2]State Key Laboratory of Organometallic Chemistry, Shanghai Institute of Organic Chemistry, Chinese Academy of Sciences, 354 Fenglin Road, Shanghai 200032, People's Republic of China

Email: Qin Xu - qinxu@ecust.edu.cn; Min Shi - Mshi@mail.sioc.ac.cn

[§]Fax: 86-21-64166128.

*Corresponding author

Experimental procedures and characterization date of compounds

CONTENTS

Spectroscopic data………………………………………………………………………………S2

References………………………………………………………………………………S14

Spectroscopic data

3a: This is a known compound.[1] A colorless oil, 31 mg, 67%; ^{1}H NMR (400 MHz, $CDCl_3$, TMS) δ 8.33-8.31 (m, 1H, ArH), 7.86-7.84 (m, 1H, ArH), 7.66 (d, J = 8.4 Hz, 1H, ArH), 7.58-7.52 (m, 2H, ArH), 7.41 (t, J = 8.0 Hz, 1H, ArH), 7.10-7.06 (m, 2H, ArH and =CH), 5.26 (d, J = 7.2 Hz, 1H, =CH), 3.81 (s, 3H, CH_3); ^{13}C NMR (100 MHz, $CDCl_3$) δ 165.3, 154.3, 153.0, 134.5, 127.5, 127.0, 126.4, 125.7, 125.3, 124.5, 121.9, 110.7, 99.8, 51.2; IR (CH_2Cl_2): ν 3056, 2949, 1714, 1646, 1507, 1438, 1391, 1261, 1190, 1151, 1103, 790, 768 cm^{-1}; MS (m/z): HRMS (ESI) Calcd. for $C_{14}H_{13}O_3$ ($[M + H]^+$): 229.0859. Found: 229.0860.

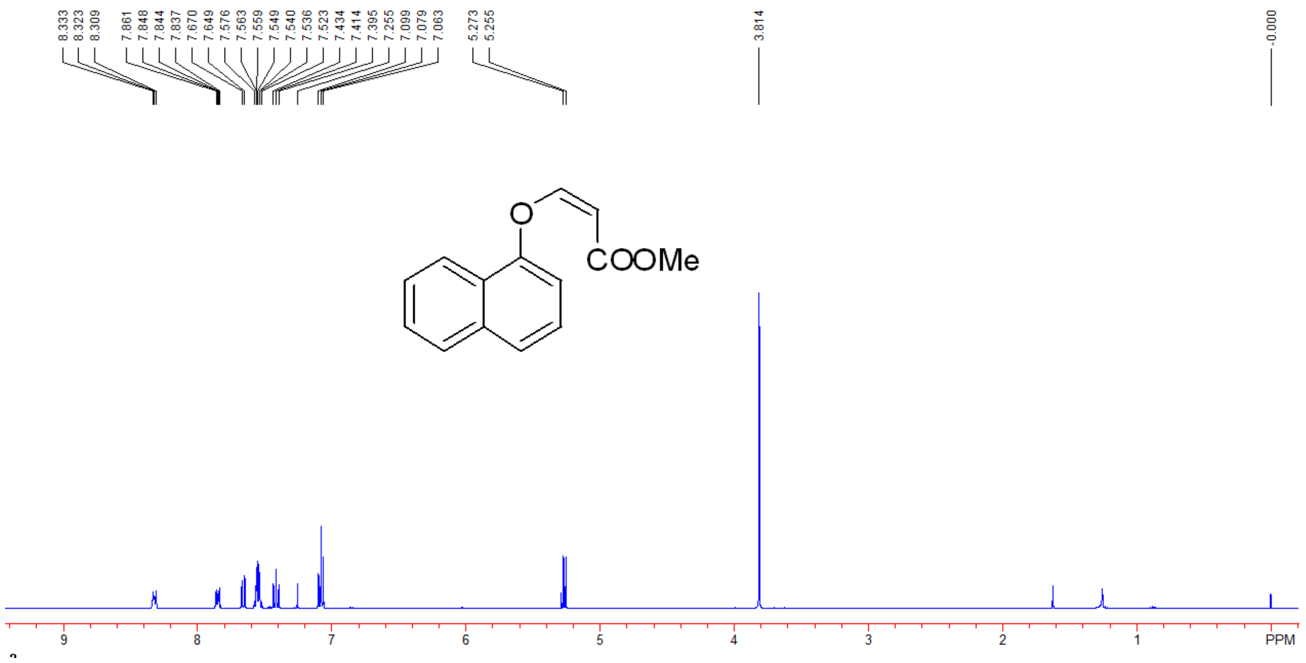

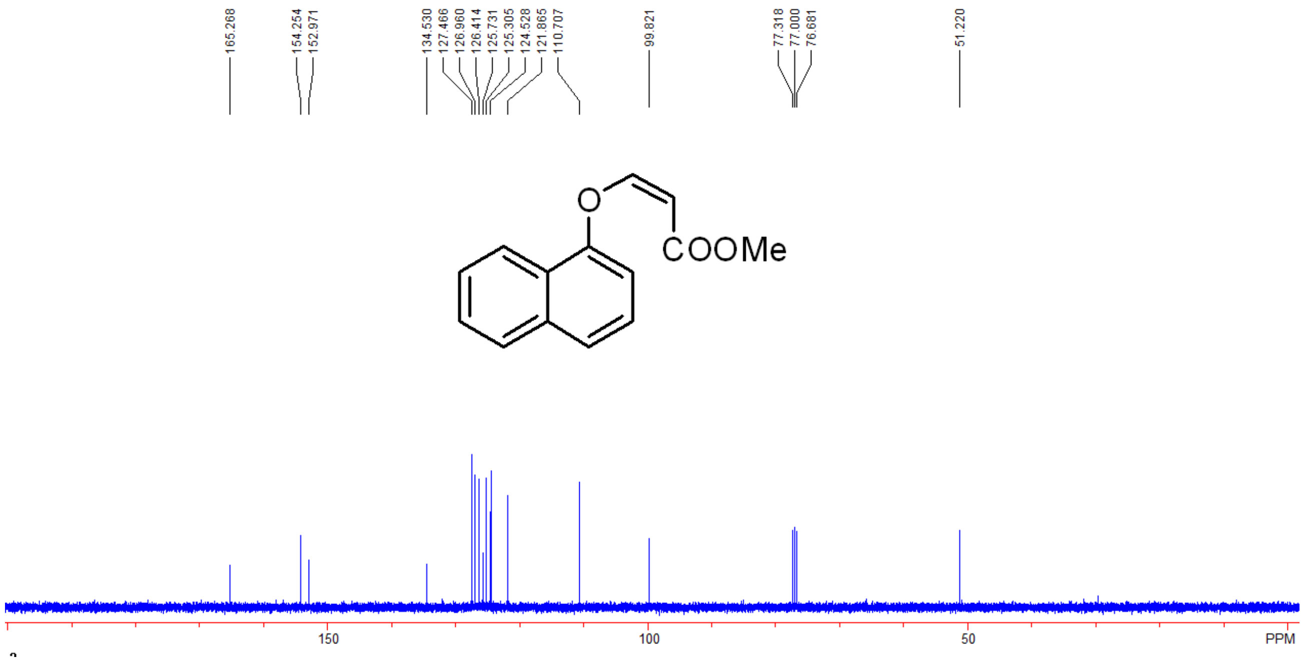

3b: This is a new compound. A colorless oil, 13 mg, 42%; ^{1}H NMR (400 MHz, $CDCl_3$, TMS) δ 8.01 (s, 1H, ArH), 7.60 (s, 1H, ArH), 7.54 (d, J = 8.8 Hz, 1H, =CH), 7.31 (t, J = 8.0 Hz, 1H, ArH), 7.02 (d, J = 7.2 Hz, 1H, =CH), 6.99 (d, J = 7.6 Hz, 1H, ArH), 5.23 (d, J = 7.2 Hz, 1H, =CH), 3.82 (s, 3H, CH_3), 2.46 (s, 3H, CH_3), 2.43 (s, 3H, CH_3); ^{13}C NMR (100 MHz, $CDCl_3$) δ 165.4, 154.9, 152.6, 136.8, 136.4, 133.6, 127.1, 124.5, 124.4, 123.7, 121.2, 110.4, 99.5, 51.2, 20.4, 20.2; IR (CH_2Cl_2): ν 2922, 1720, 1647, 1573, 1454, 1434, 1265, 1191, 1161, 1141, 1096, 790, 743 cm^{-1}; MS (m/z): HRMS (ESI) Calcd. for $C_{16}H_{17}O_3$ ($[M + H]^+$): 257.1172. Found: 257.1168.

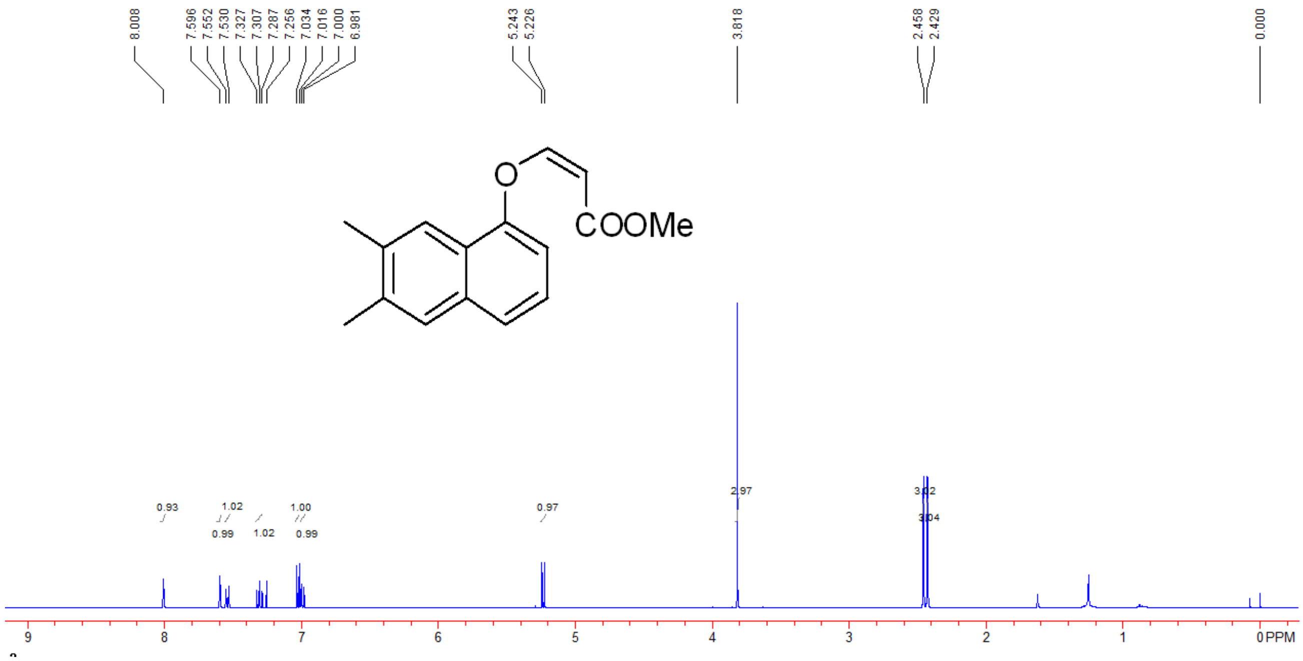

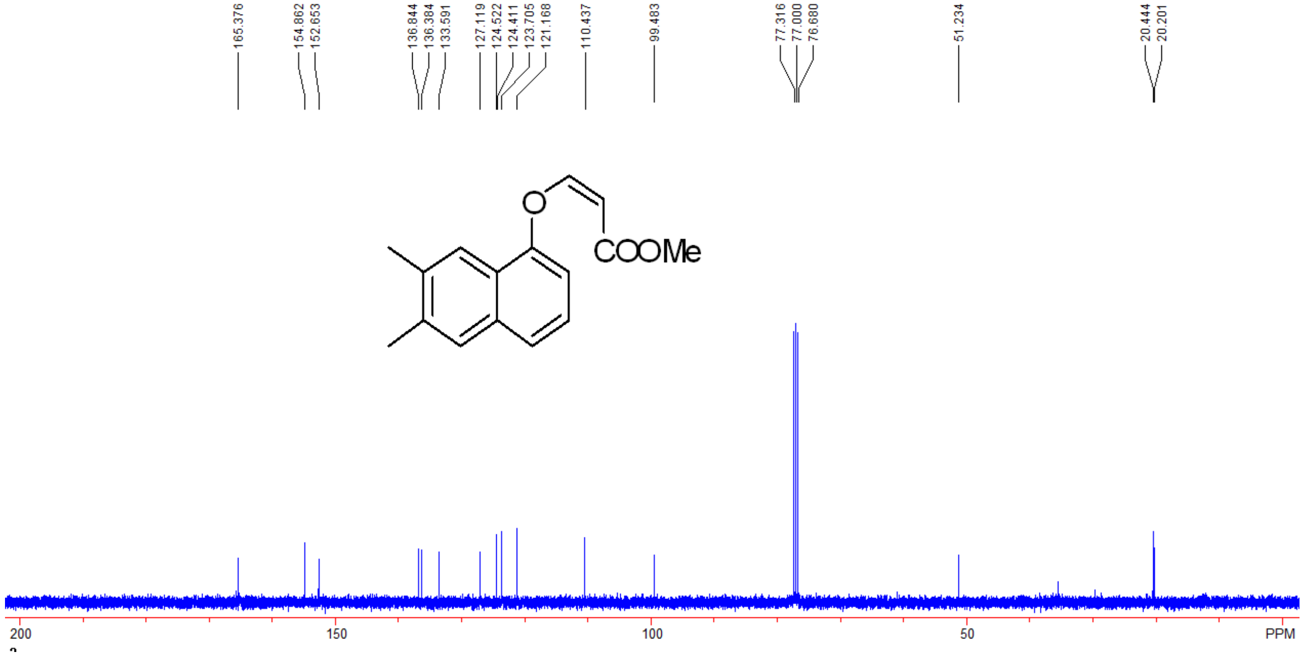

3c: This is a new compound. A colorless oil, 35 mg, 66%; ^{1}H NMR (400 MHz, $CDCl_3$, TMS) δ 8.09-8.04 (m, 1H, ArH), 7.59-7.54 (m, 2H, ArH), 7.42 (t, J = 8.0 Hz, 1H, ArH), 7.08-7.06 (m, 2H, ArH and =CH), 5.31 (d, J = 6.8 Hz, 1H, =CH), 3.82 (s, 3H, CH_3); ^{19}F NMR (376 MHz, $CDCl_3$, $CFCl_3$) δ -135.5- -135.6 (m), -135.9- -136.0 (m); ^{13}C NMR (100 MHz, $CDCl_3$) δ 165.0, 153.1, 152.4, 152.34, 152.31, 152.29, 152.1, 151.9, 151.6, 151.4, 149.6, 149.4, 149.1, 148.8, 131.6, 131.55, 131.49, 131.48, 126.04, 126.03, 126.01, 123.52, 123.50, 123.47, 123.4, 122.64, 122.63, 122.56, 122.55, 113.53, 113.52, 113.36,

113.35, 110.35, 110.34, 110.33, 108.93, 108.92, 108.75, 108.73, 100.8, 51.3; IR (CH_2Cl_2): ν 2952, 1717, 1647, 1519, 1471, 1459, 1268, 1187, 1164, 1142, 867, 804, 787, 739 cm^{-1}; MS (*m*/*z*): HRMS (ESI) Calcd. for $C_{14}H_{11}F_2O_3$ ($[M + H]^+$): 265.0671. Found: 265.0667.

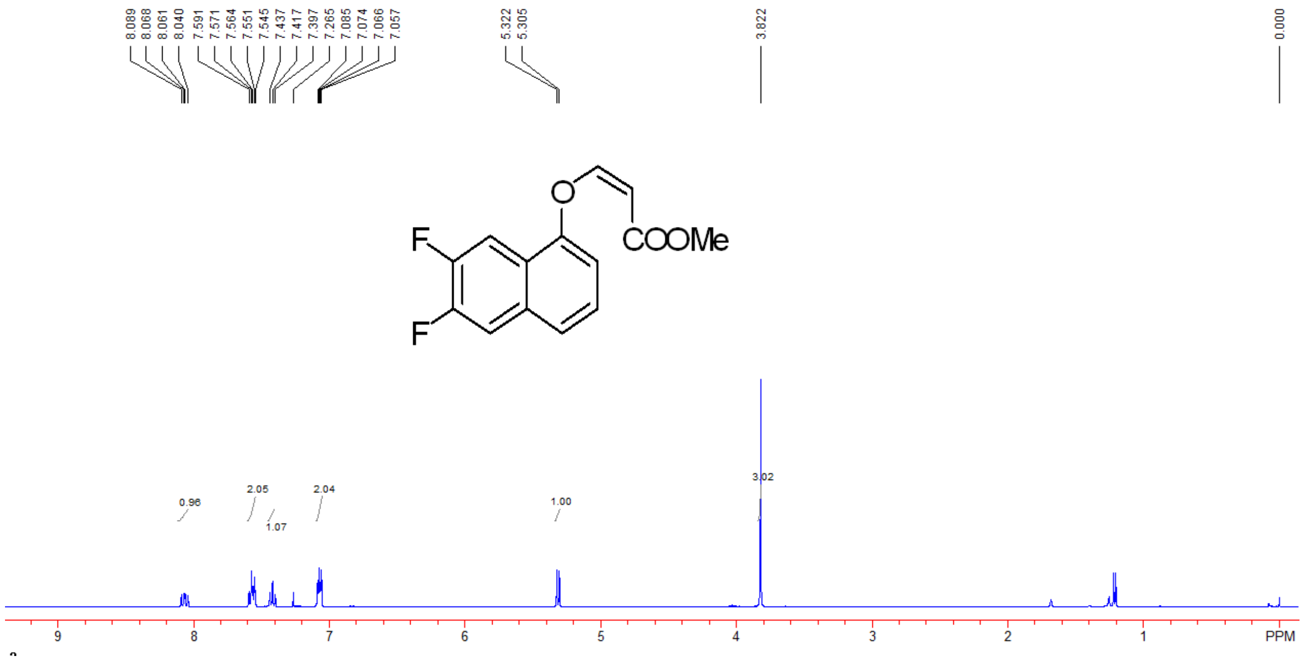

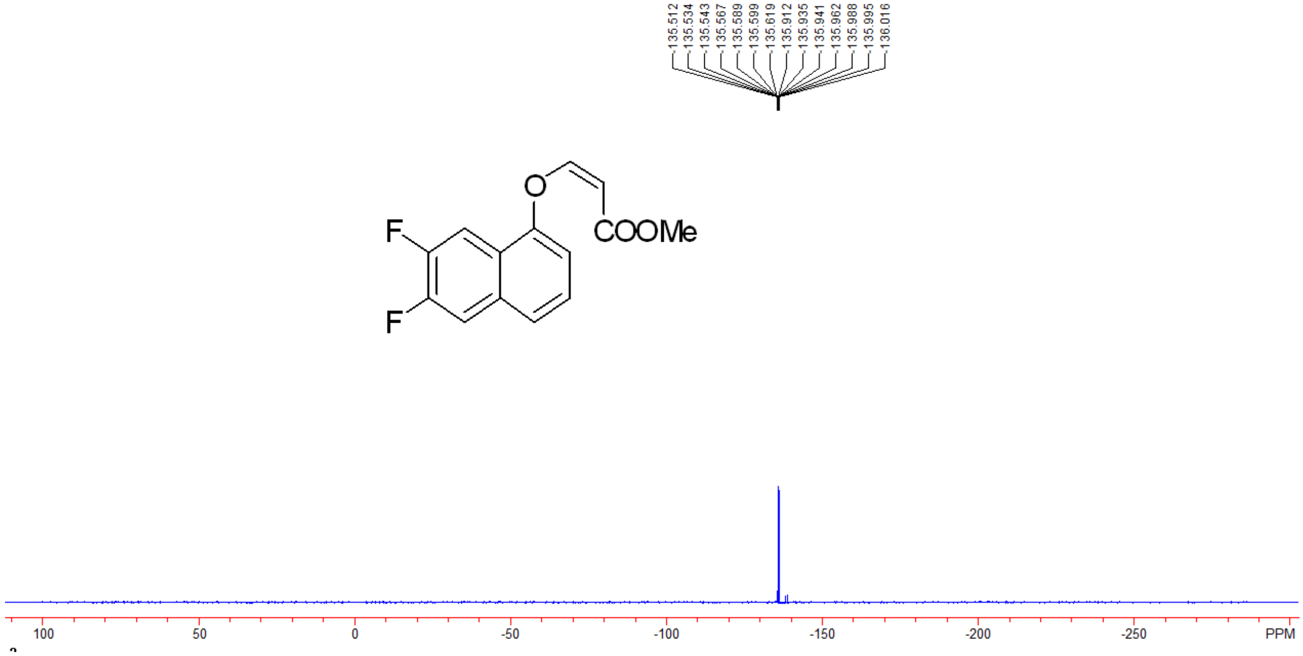

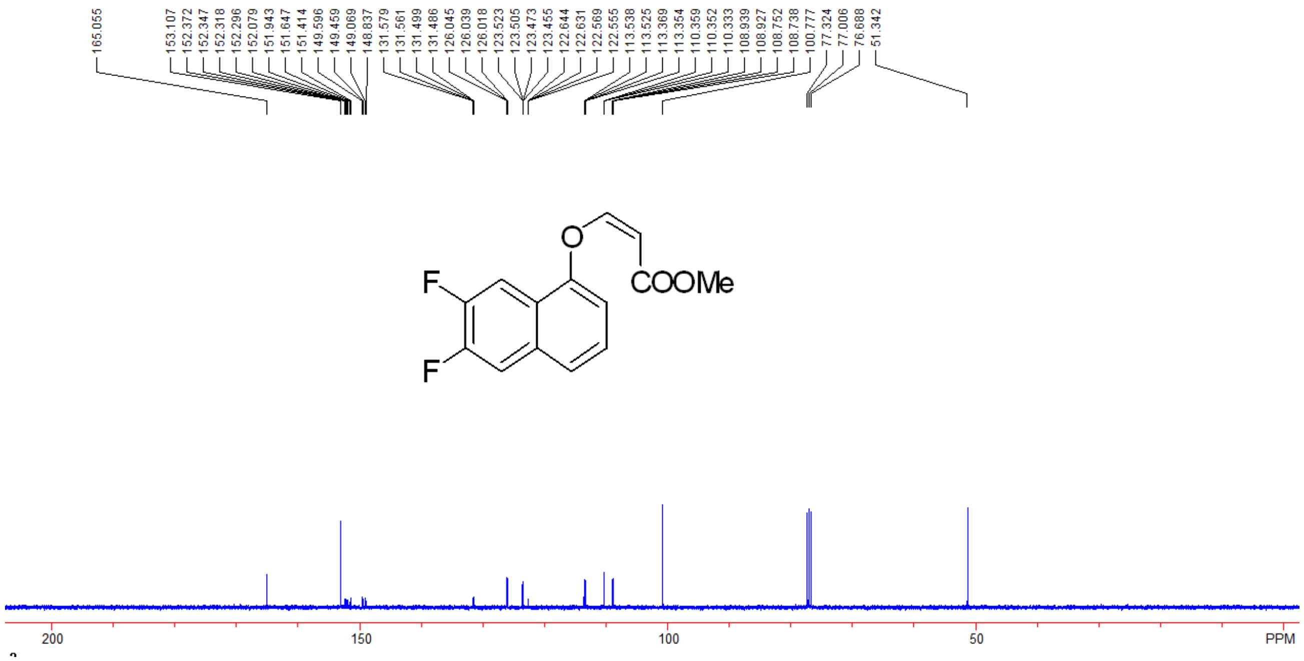

3d: This is a new compound. A colorless oil, 58 mg, 76%; ^{1}H NMR (400 MHz, $CDCl_3$, TMS) δ 8.60 (s, 1H, ArH), 8.15 (s, 1H, ArH), 7.53 (d, J = 8.4 Hz, 1H, ArH), 7.47-7.44 (m, 1H, ArH), 7.10 (dd, J_1 = 7.2 Hz, J_2 = 0.8 Hz, 1H, ArH), 7.04 (d, J = 6.8 Hz, 1H, =CH), 5.33 (d, J = 6.8 Hz, 1H, =CH), 3.83 (s, 3H, CH_3); ^{13}C NMR (100 MHz, $CDCl_3$) δ 165.0, 152.8, 151.8, 134.1, 131.8, 127.0, 126.9, 125.3, 123.7, 123.1, 122.9, 111.3, 101.2, 51.4; IR (CH_2Cl_2): ν 2945, 2924, 2853, 1714, 1651, 1567, 1436, 1418, 1259, 1191, 1159, 1102, 783, 737 cm^{-1}; MS (m/z): HRMS (ESI) Calcd. for $C_{14}H_{11}Br_2O_3$ ($[M + H]^+$): 384.9069. Found: 384.9054.

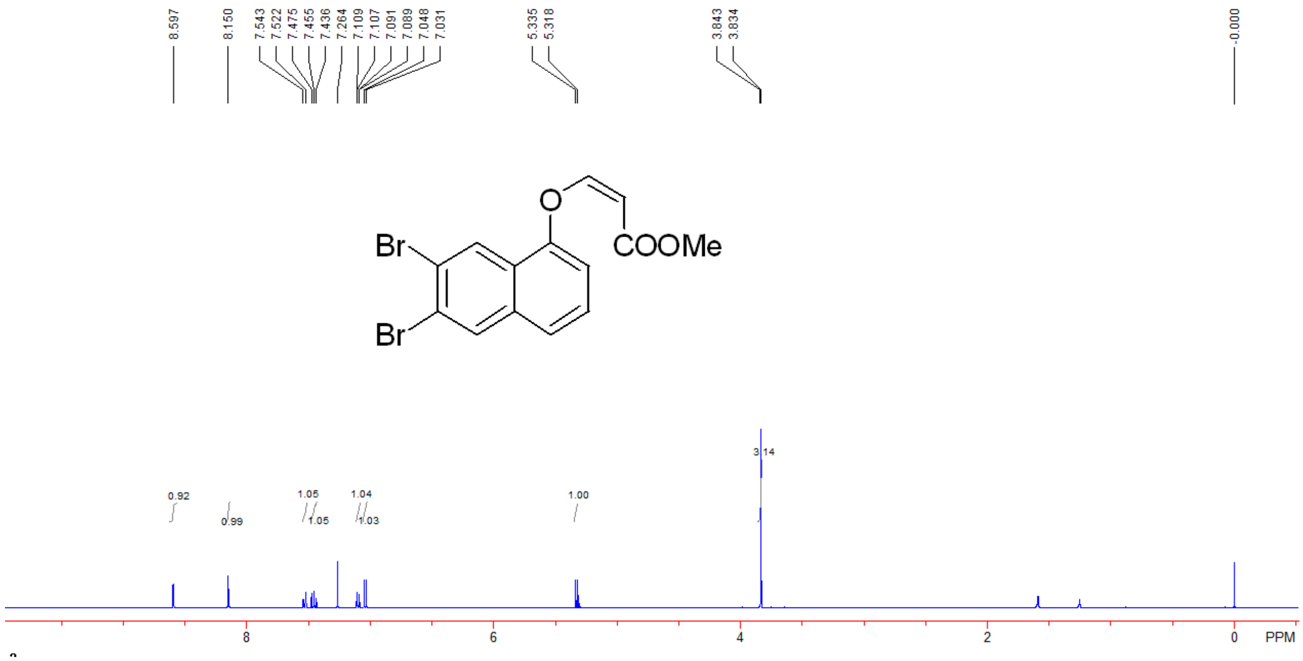

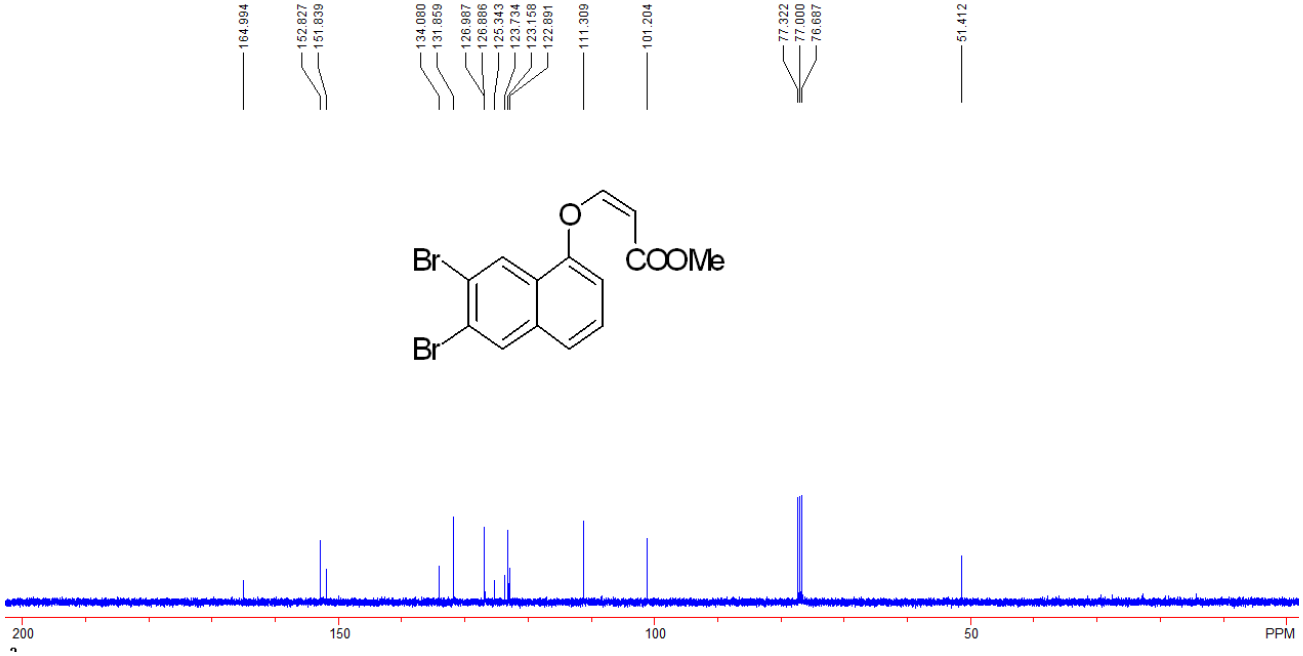

3e: This is a known compound.[2] A colorless oil, 26 mg, 54%; ^{1}H NMR (400 MHz, $CDCl_3$, TMS) δ 8.35-8.32 (m, 1H, ArH), 7.86-7.84 (m, 1H, ArH), 7.66 (d, *J* = 8.4 Hz, 1H, ArH), 7.58-7.52 (m, 2H, ArH), 7.42 (t, *J* = 8.0 Hz, 1H, ArH), 7.10-7.07 (m, 2H, ArH and =CH), 5.25 (d, *J* = 6.8 Hz, 1H, =CH), 4.28 (q, *J* = 7.2 Hz, 2H, CH_2), 1.36 (t, *J* = 7.2 Hz, 3H, CH_3); ^{13}C NMR (100 MHz, $CDCl_3$) δ: 164.9, 153.9, 153.0, 134.6, 127.5, 127.0, 126.4, 125.8, 125.3, 124.5, 122.0, 110.5, 100.4, 60.0, 14.4; IR

(CH_2Cl_2): ν 3057, 2976, 1716, 1645, 1576, 1507, 1392, 1261, 1154, 1106, 795, 771 cm^{-1}; MS (*m*/*z*): HRMS (ESI) Calcd. for $C_{15}H_{15}O_3$ ($[M + H]^+$): 243.1016. Found: 243.1014.

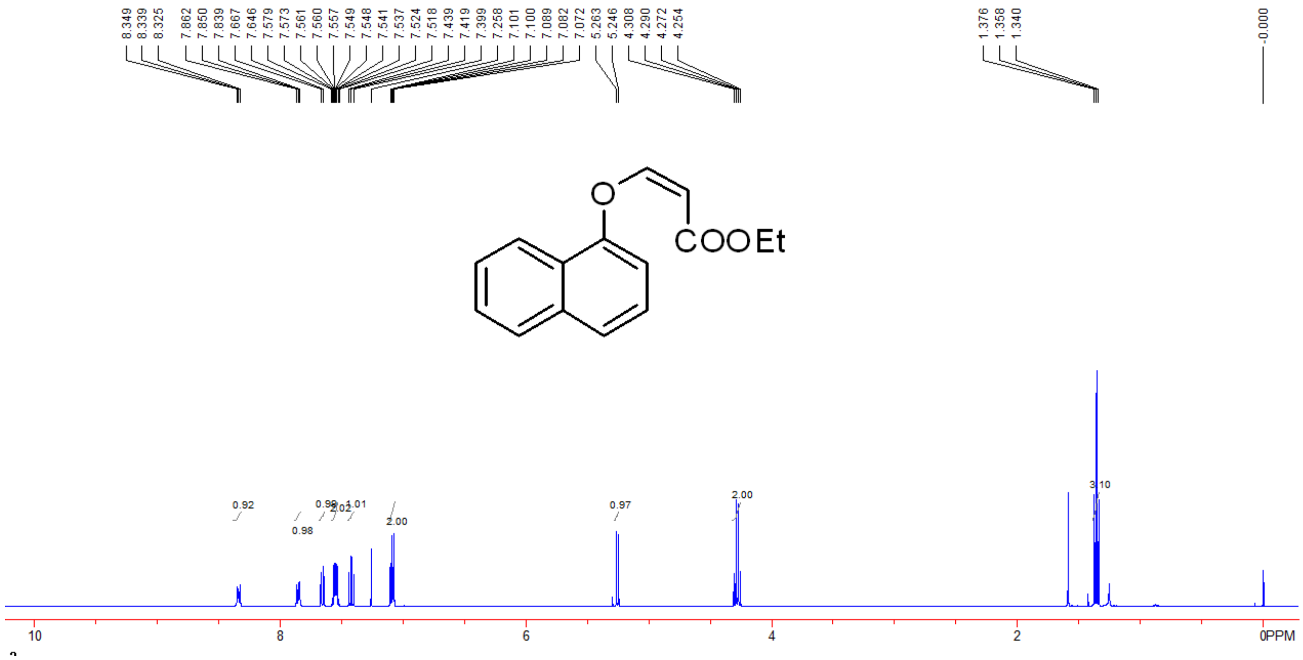

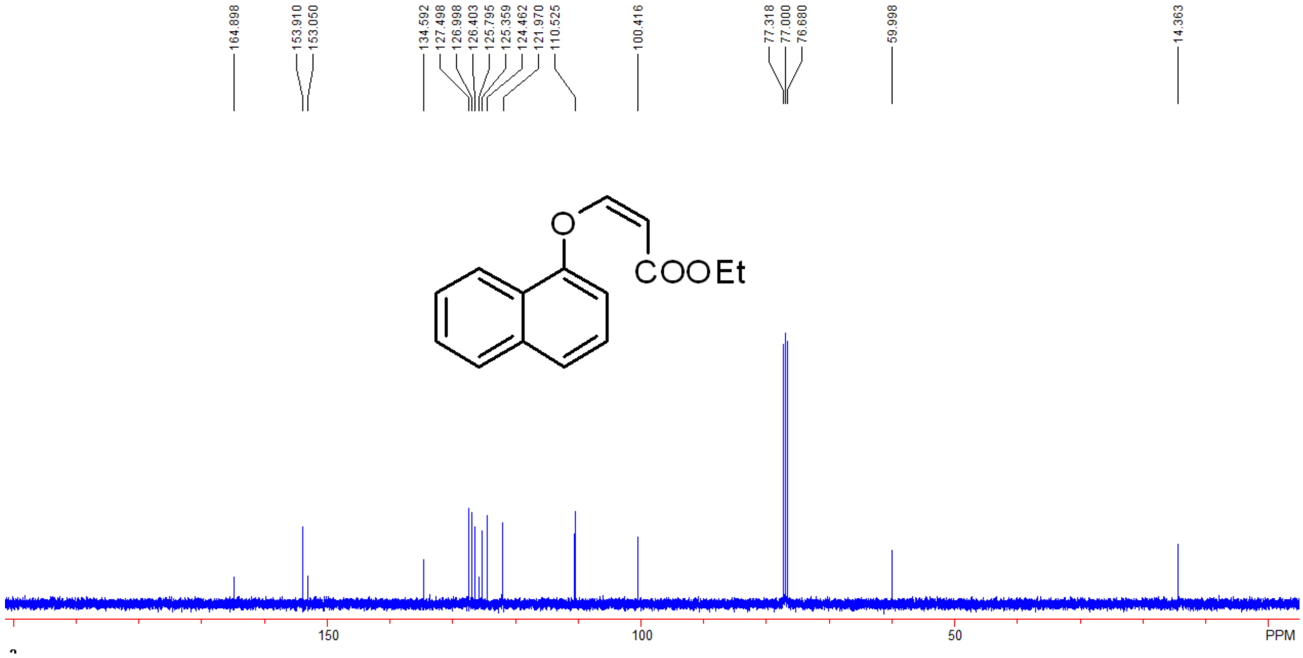

3f: This is a new compound. A colorless oil, 45 mg, 84%; ^{1}H NMR (400 MHz, $CDCl_3$, TMS) δ 8.34-8.32 (m, 1H, ArH), 7.85-7.82 (m, 1H, ArH), 7.63 (d, *J* = 8.4 Hz, 1H, ArH), 7.56-7.51 (m, 2H, ArH), 7.40 (t, *J* = 8.0 Hz, 1H, ArH), 7.06 (d, *J* = 7.6 Hz, 1H, ArH), 6.96 (d, *J* = 6.8 Hz, 1H, =CH), 5.16 (d, *J* =

6.8 Hz, 1H, =CH), 1.56 (s, 9H, CH_3); ^{13}C NMR (100 MHz, $CDCl_3$) δ 164.2, 153.2, 153.1, 134.5, 127.4, 126.9, 126.3, 125.8, 125.4, 124.3, 122.0, 110.7, 102.3, 80.2, 28.3; IR (CH_2Cl_2): ν 2976, 2930, 1713, 1695, 1641, 1391, 1366, 1262, 1206, 1131, 1099, 794, 770 cm^{-1}; MS (*m*/*z*): HRMS (ESI) Calcd. for $C_{17}H_{18}NaO_3$ ($[M + Na]^+$): 293.1148. Found: 293.1152.

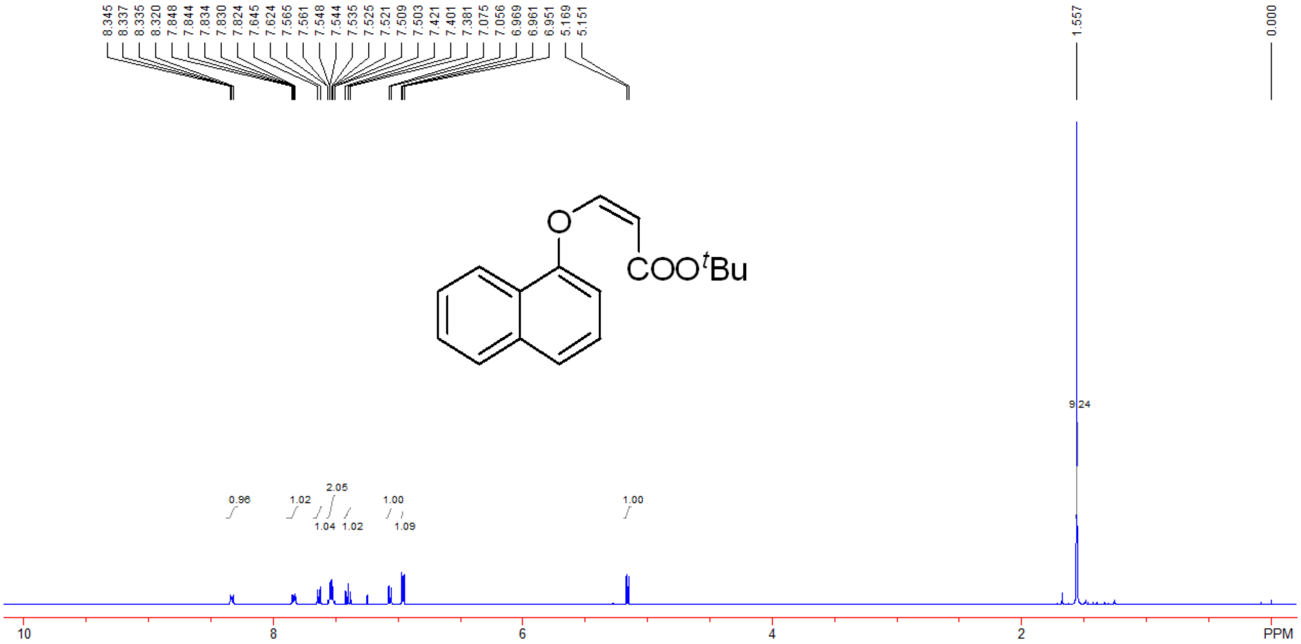

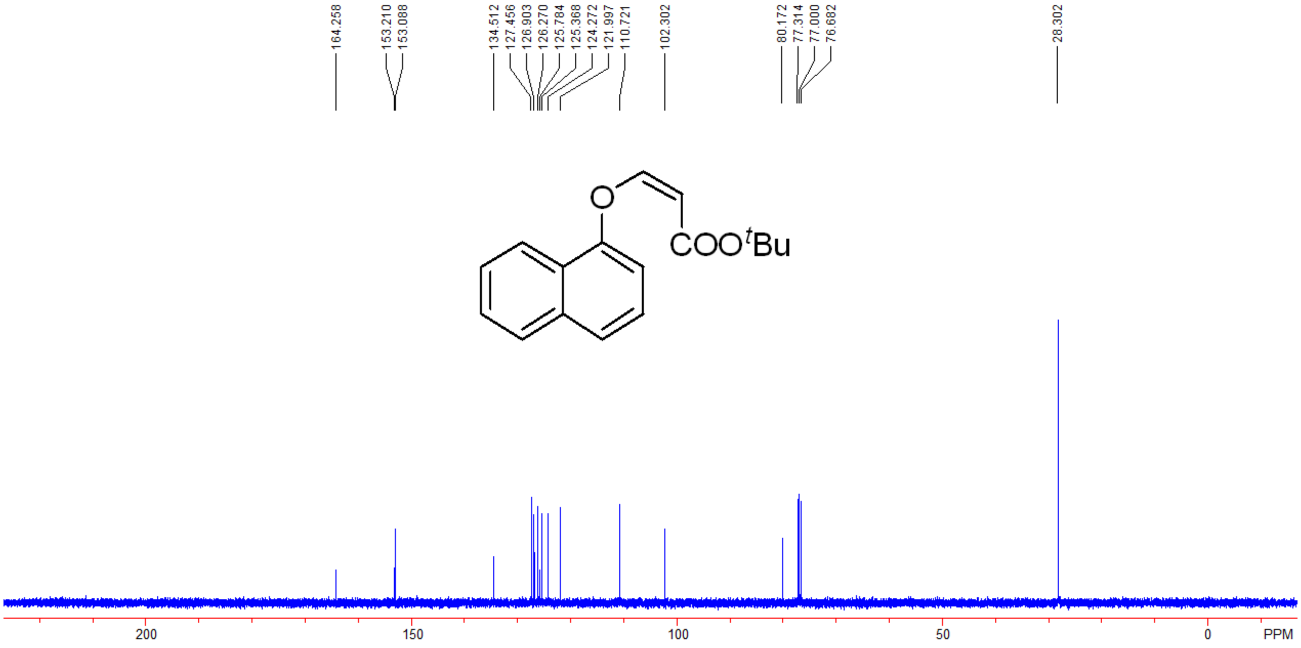

3g: This is a new compound. A colorless oil, 35 mg, 58%; ^{1}H NMR (400 MHz, $CDCl_3$, TMS) δ 8.03 (s, 1H, ArH), 7.59 (s, 1H, ArH), 7.53 (d, J = 8.4 Hz, 1H, ArH), 7.31 (t, J = 8.0 Hz, 1H, ArH), 6.98 (d, J = 7.2 Hz, 1H, ArH), 6.94 (d, J = 7.2 Hz, 1H, =CH), 5.13 (d, J = 7.2 Hz, 1H, =CH), 2.46 (s, 3H, CH_3), 2.43 (s, 3H, CH_3), 1.57 (s, 9H, CH_3); ^{13}C NMR (100 MHz, $CDCl_3$) δ 164.5, 153.6, 152.8, 136.7, 136.2, 133.6, 127.1, 124.56, 124.46, 123.4, 121.3, 110.3, 102.0, 80.2, 28.3, 20.3, 20.2; IR (CH_2Cl_2): ν 2975, 2923, 2854, 1716, 1698, 1645, 1434, 1366, 1264, 1207, 1137, 1081, 903, 741 cm^{-1}; MS (m/z): HRMS (ESI) Calcd. for $C_{19}H_{22}NaO_3$ ($[M + Na]^+$): 321.1461. Found: 321.1462.

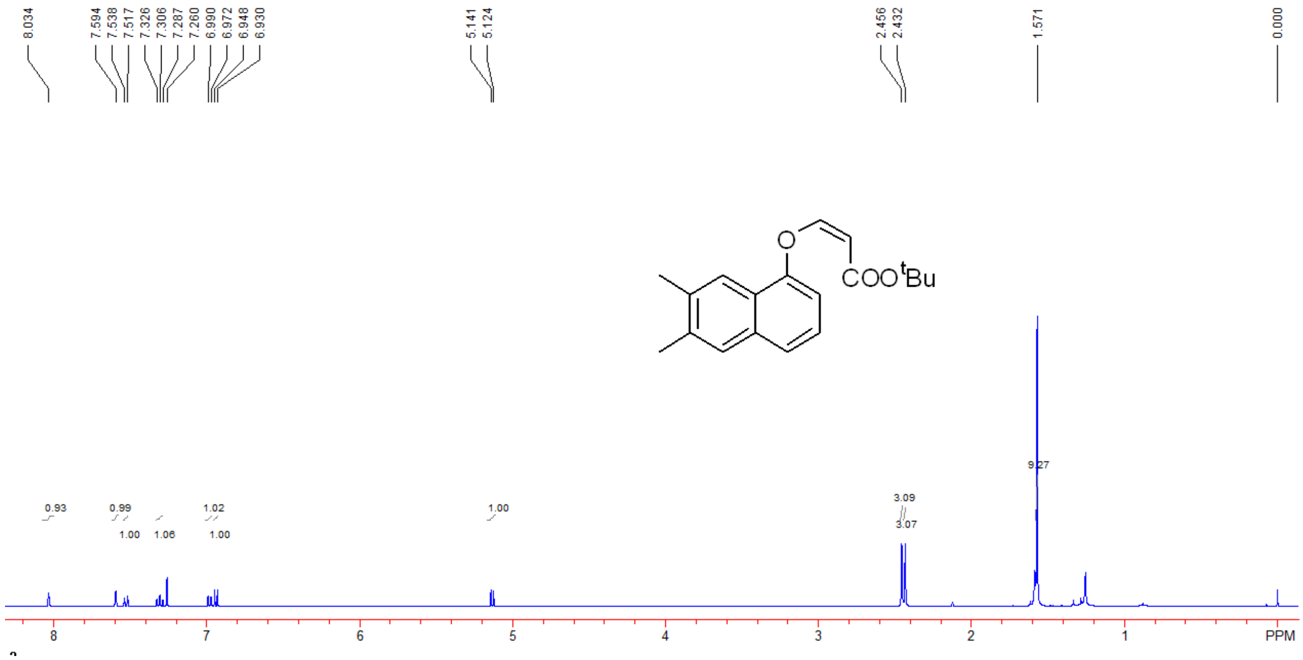

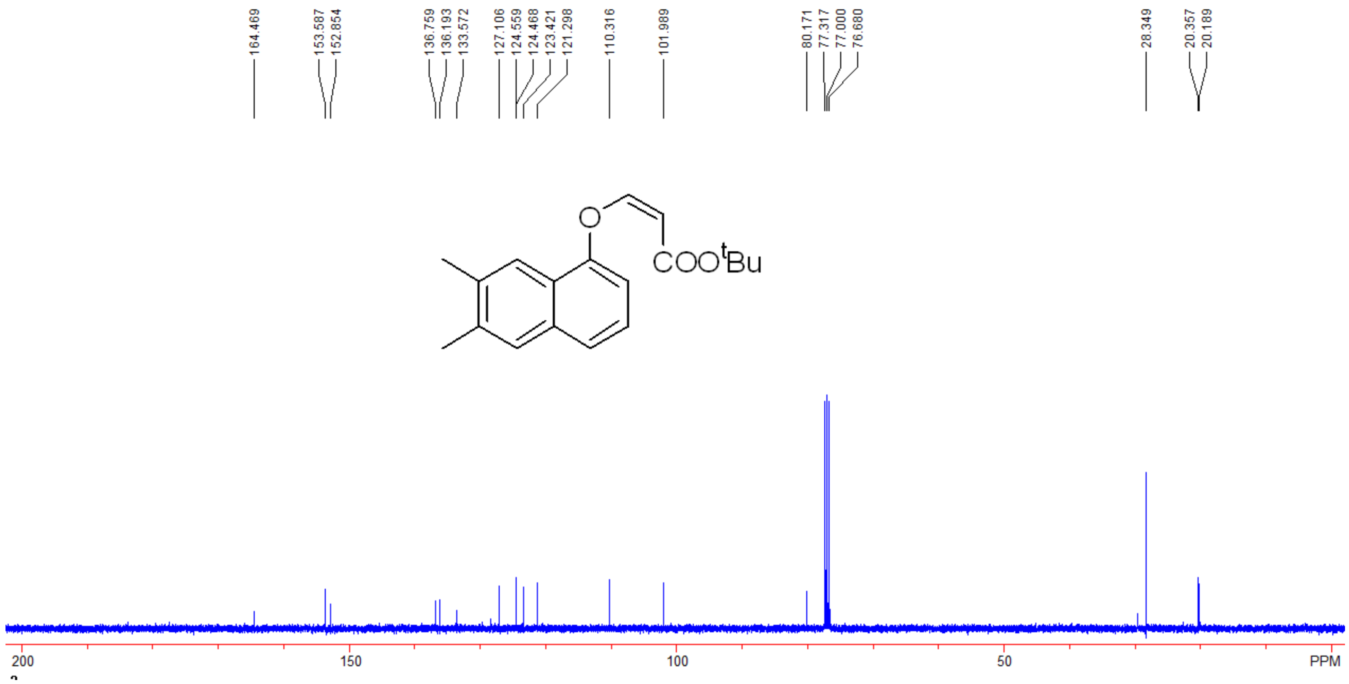

3h: This is a new compound. A colorless oil, 61 mg, 72%; ^{1}H NMR (400 MHz, $CDCl_3$, TMS) δ 8.61 (s, 1H, ArH), 8.13 (s, 1H, ArH), 7.51 (d, J = 8.4 Hz, 1H, ArH), 7.44 (t, J = 8.0 Hz, 1H, ArH), 7.07 (d, J = 7.2 Hz, 1H, ArH), 6.94 (d, J = 6.8 Hz, 1H, =CH), 5.21 (d, J = 6.8 Hz, 1H, =CH), 1.57 (s, 9H, CH_3); ^{13}C NMR (100 MHz, $CDCl_3$) δ 164.0, 152.0, 151.5, 134.1, 131.9, 127.1, 126.9, 125.4, 123.6, 122.8, 122.7, 111.2, 103.7, 80.6, 28.3 cm^{-1}; IR (CH_2Cl_2): ν 2976, 2927, 1696, 1645, 1568, 1418, 1366, 1259, 1207, 1140, 1104, 872, 785, 737 cm^{-1}; MS (*m*/*z*): HRMS (ESI) Calcd. for $C_{13}H_9Br_2O_3$ ([M + H – *t*-Bu]$^+$): 370.8913. Found: 370.8915.

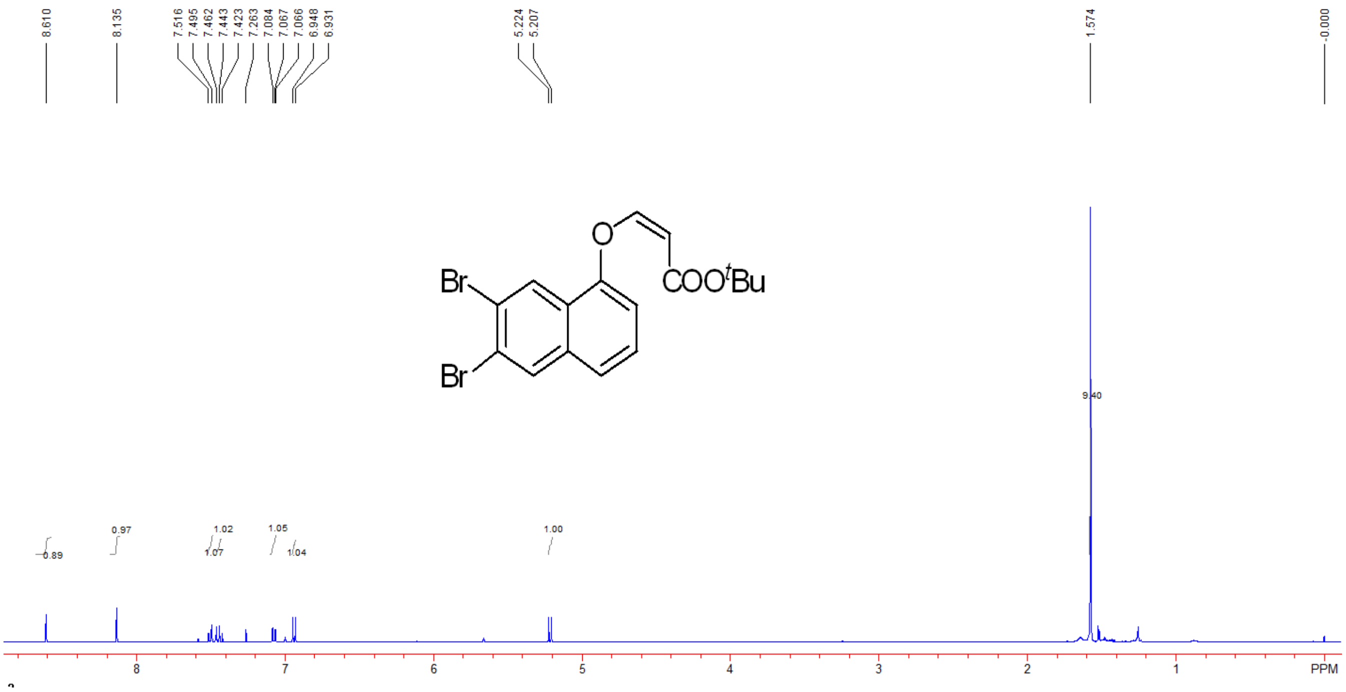

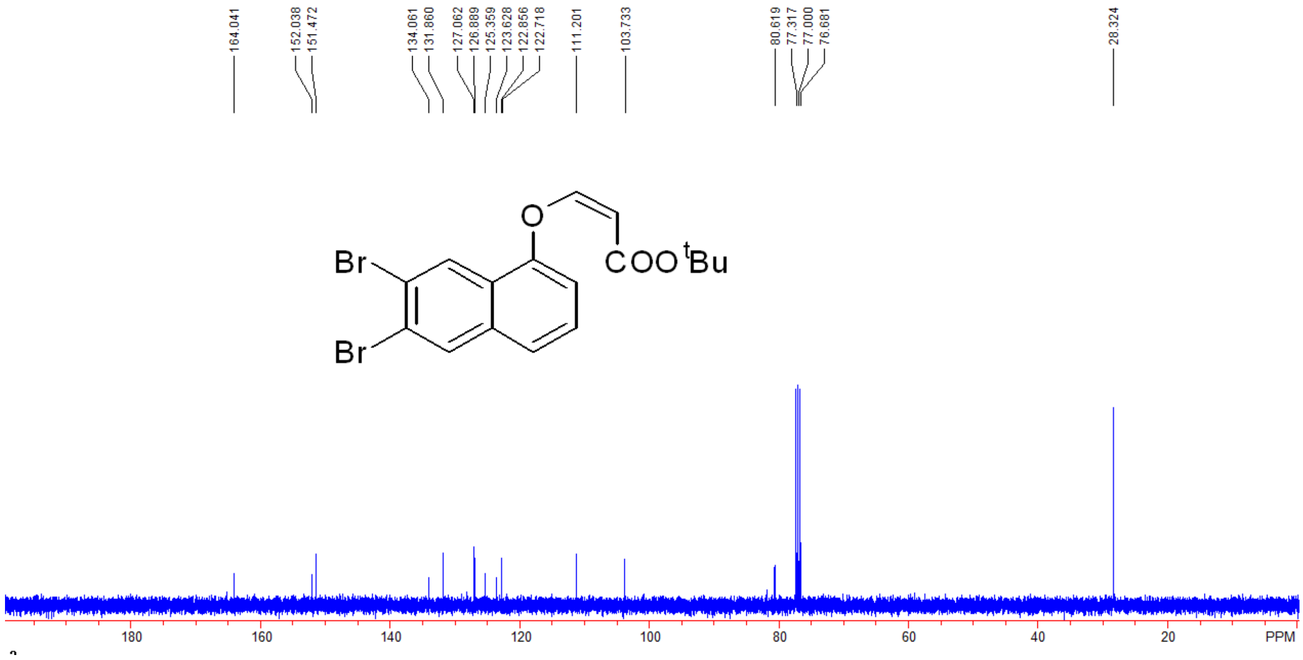

3i: This is a known compound.[3] A colorless oil, 20 mg, 48%; ^{1}H NMR (400 MHz, $CDCl_3$, TMS) δ 8.08-8.06 (m, 1H, ArH), 7.90-7.87 (m, 2H, ArH), 7.71 (d, *J* = 8.4 Hz, 1H, ArH), 7.57-7.54 (m, 2H, ArH and =CH), 7.45 (t, *J* = 8.0 Hz, 1H, ArH), 7.14 (d, *J* = 7.2 Hz, 1H, ArH), 5.99 (d, *J* = 12.8 Hz, 1H, =CH), 2.25 (s, 3H, CH_3); ^{13}C NMR (100 MHz, $CDCl_3$) δ 197.3, 159.7, 151.7, 134.7, 127.8, 127.0, 126.6, 125.7, 125.5, 125.3, 121.3, 113.0, 111.9, 28.3; IR (CH_2Cl_2): ν 3056, 2924, 1638, 1612, 1574, 1391,

1229, 1179, 1160, 1133, 951, 795, 770, 729 cm^{-1}; MS (m/z): HRMS (ESI) Calcd. for $C_{14}H_{13}O_2$ ([M + H]$^+$): 213.0910. Found: 213.0902.

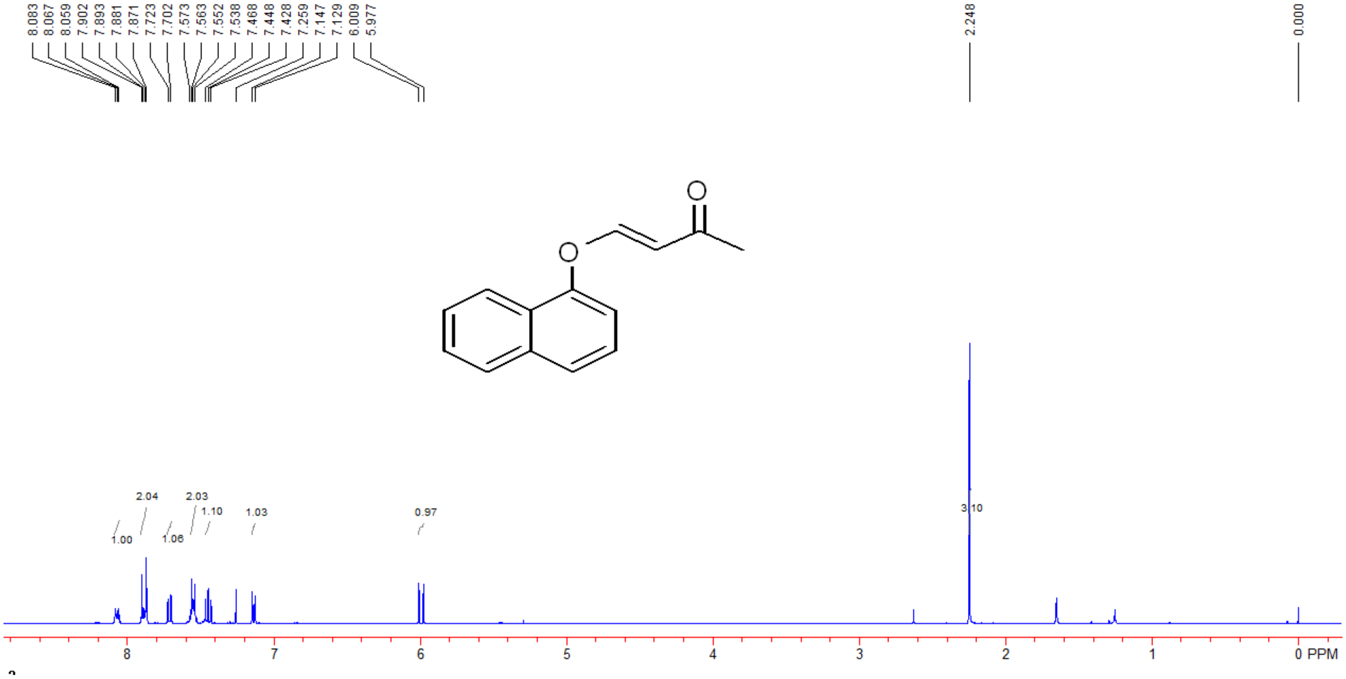

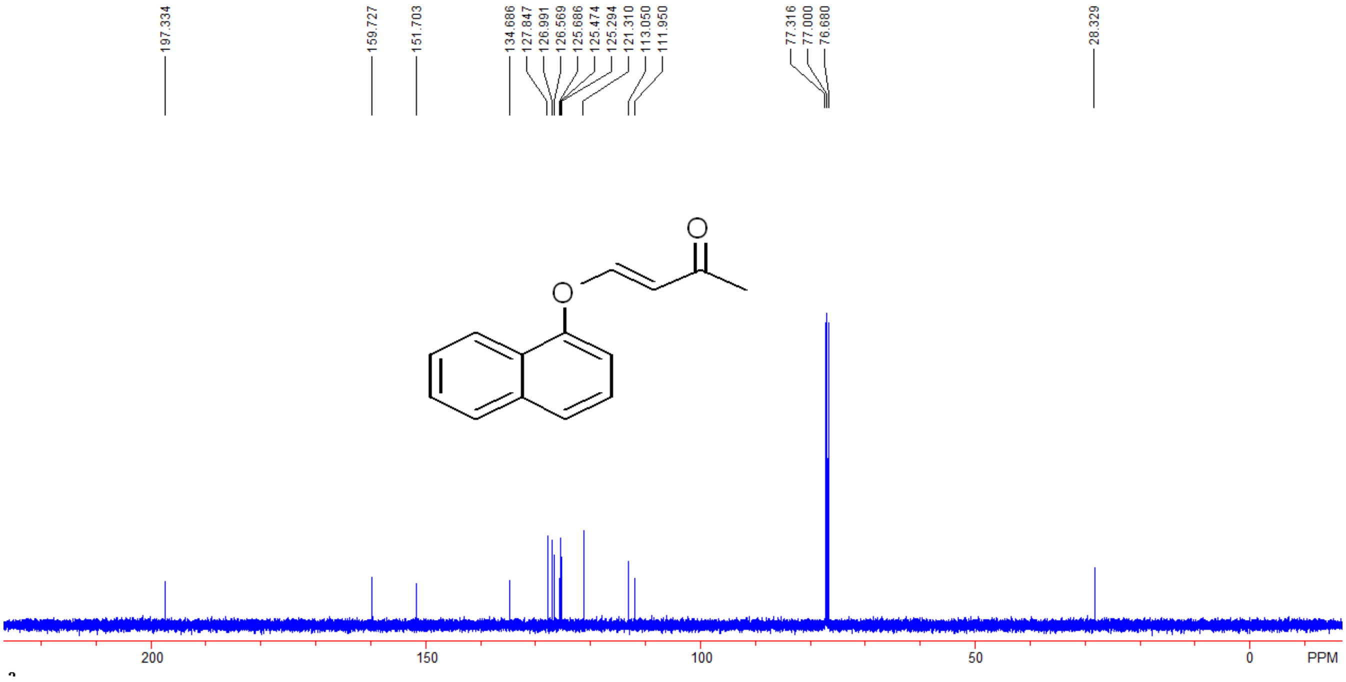

References

1. Nasiri, F.; Malakutikhah, D. *Monatsh. Chem.* **2011**, *142*, 807–812. doi:10.1007/s00706-011-0514-6
2. Kabir, M. S.; Namjoshi, O. A.; Verma, R.; Lorenz, M.; Phani Babu Tiruveedhula, V. V. N.; Monte, A.; Bertz, S. H.; Schwabacher, A. W.; Cook, J. M. *J. Org. Chem.* **2012**, *77*, 300–310. doi:10.1021/jo201948e
3. Kochetkov, N. K.; Rybinskaya, M. I.; Nesmeyanov, A. N. *Doklady Akademii Nauk* **1951**, *79*, 799–802.
